# Impact of Perinatal Exposure to SARS-CoV-2 Infection on Early Health Outcomes among Infants Born from 2020 to 2021 in British Columbia, Canada

**DOI:** 10.1155/2023/9968774

**Published:** 2023-05-05

**Authors:** Lindsay L. Richter, Matthew S. P. Ho, Qian Zhang, Jeffrey N. Bone, Elodie Portales-Casamar, Connie L. Yang, Ashley Roberts, Kristopher Kang, Emily Kieran, Carol Lam, Sarka Lisonkova, Joseph Y. Ting

**Affiliations:** ^1^Department of Pediatrics, University of British Columbia, Vancouver, BC, Canada; ^2^Department of Pediatrics, University of Alberta, Edmonton, AB, Canada; ^3^Research Informatics, BC Children's Hospital Research Institute, Vancouver, BC, Canada; ^4^Department of Obstetrics and Gynaecology, University of British Columbia, Vancouver, BC, Canada; ^5^Department of Pediatrics, University of Toronto, Toronto, ON, Canada

## Abstract

**Background:**

The severe acute respiratory syndrome coronavirus 2 (SARS-CoV-2) pandemic has impacted healthcare services and outcomes. We aimed to investigate healthcare resource utilization and early health outcomes of infants born to mothers with perinatal SARS-CoV-2 infection.

**Methods:**

The study included all infants born alive between February 1, 2020, and April 30, 2021, in British Columbia. We used linked provincial population-based databases including data on COVID-19 testing, birth, and health information for up to one year from birth. Perinatal COVID-19 exposure for infants was defined being born to mothers with a positive test for SARS-CoV-2 infection during pregnancy or at delivery. Cases of COVID-19-exposed infants were matched with up to four non‐exposed infants by birth month, sex, birthplace, and gestational age in weeks. Outcomes included hospitalizations, emergency department visits, and in-/outpatient diagnoses. Outcomes were compared between groups using conditional logistic regression and linear mixed effects models including effect modification by maternal residence.

**Results:**

Among 52,711 live births, 484 infants had perinatal exposure to SARS-CoV-2, an incidence rate of 9.18 per 1000 live births. Exposed infants (54.6% male) had a mean gestational age of 38.5 weeks, and 99% were born in hospital. Proportions of infants requiring at least one hospitalization (8.1% vs. 5.1%) and at least one emergency department visit (16.9% vs. 12.9%) were higher among the exposed vs. unexposed infants, respectively. Among infants from the urban area, those with exposure were more likely to have respiratory infectious diseases (odds ratio: 1.74; 95% confidence intervals: 1.07, 2.84), compared with those without exposure. *Interpretation*. In our cohort, infants born to mothers with SARS-CoV-2 infection have increased healthcare demands in their early infancy, which warrants further investigation.

## 1. Introduction

The severe acute respiratory syndrome coronavirus 2 (SARS-CoV-2) has caused six million deaths globally in the past two years of the pandemic [1]. In Canada, the pandemic has resulted in over 3.6 million infected cases and 38,000 associated deaths [2]. Vulnerable groups such as pregnant women, in which SARS-CoV-2 viral infection may cause complications not only to the pregnant woman but also to the fetus, may be disproportionately impacted. SARS-CoV-2 infection can lead to an elevated risk of adverse pregnancy outcomes including preterm delivery [3, 4] and thus increased healthcare utilization and resource demands.

The impact of perinatal exposure to SARS-CoV-2 infection on fetal development and subsequent infant health is not well understood. Several papers have studied the outcomes of neonates born to SARS-CoV-2-positive mothers from 2021 to 2022, including cohort studies from the United States [5–10], Italy [11], and Sweden [12]. The results of these studies showed that the rate of perinatal infection among neonates as a result of maternal positivity for SARS-CoV-2 was approximately 2.2% [5]. Most neonates were asymptomatic [6] and if symptomatic, the symptoms were minimal [7]. None had serious adverse outcomes [10, 12]. In terms of follow-up of these infants, only two papers have described neurodevelopmental status at six or ten–twelve months of age [8, 13] and showed that in utero exposure to maternal SARS-CoV-2 infection was not associated with neurodevelopmental impairment. To our knowledge, there have been no other long-term follow-up studies examining infant health outcomes and healthcare resource utilization to date.

To address this knowledge gap in the literature, we conducted a retrospective cohort study to compare infants born to mothers with positive SARS-CoV-2 infection during pregnancy or at delivery with infants born to mothers without such infection. Our aim was to investigate health outcomes and healthcare resource utilization during the first year of life among the infants with perinatal exposure to SARS-CoV-2.

## 2. Methods

### 2.1. Data Sources

The birth records of all liveborn infants in British Columbia (BC), Canada, between February 1, 2020, and April 30, 2021, were obtained from Population Data BC (PopData BC) [14]. To define the birth cohort, we used the Vital Statistics Births dataset for hospital and out-of-hospital births linked with COVID-19 testing data [15, 16]. Four administrative population-based databases were used to obtain the longitudinal health data of the infants throughout their first year of life (0–12-month follow-up after birth) including (1) Discharge Abstract Database (DAD) which contains hospitalization information with up to 26 diagnostic and procedure codes; (2) Medical Services Plan (MSP) Payment Information which contains data on medical services provided by fee-for-service practitioners and outpatient consultations; (3) National Ambulatory Care Reporting System (NACRS) which contains data on emergency department (ED) visits; and (4) PharmaNet which contains information on all filled prescriptions at community and outpatient pharmacies [17–20].

### 2.2. Outcome Measures

The primary outcomes included ED visits, hospitalizations, and composite outcomes of adverse early health outcomes including allergic diseases, endocrine disorders, renal conditions, respiratory conditions, and infectious diseases. The secondary outcomes included reasons for ED visit or hospitalization, individual in-/outpatient diagnoses, and outpatient medications. Diagnoses were identified using the International Classification of Diseases 9^th^ Edition (ICD-9) from MSP data (outpatient visit) or 10^th^ Edition (ICD-10) from DAD data (inpatient hospitalizations). Diagnoses and intervention codes are listed in Supplementary Table 1. PharmaNet prescription medications were identified by their Drug Identification Number (DIN) and classification using the PharmaCare Theraclass system [20]. Medications are listed in Supplementary Table 2.

### 2.3. Exposure and Effect Modifier

Infant health data were linked to the maternal SARS-CoV-2 PCR testing data. A cohort of infants with perinatal SARS-CoV-2 exposure (exposed cohort) was defined as those born to mothers with a positive SARS-CoV-2 infection during pregnancy or at delivery. The non‐exposed cohort included infants born to mothers with negative SARS-CoV-2 results or to mothers without a test for SARS-CoV-2. Infants in the exposed cohort were compared with up to four infants in the non‐exposed cohort matched by birth month, sex, place of birth (hospital vs. home), and gestational age (±2 weeks). To evaluate any difference between rural and urban areas in the access of healthcare resources, residence areas according to maternal postal codes from the birth records were treated as an effect modifier, and the effect of SARS-CoV-2 exposure was examined by stratification of rural vs. urban areas.

### 2.4. Statistical Analysis

The sample size was determined by the number of all cases of SARS-CoV-2-exposed infants as well as number of matched controls (ratio 1 : 4) in BC during the study period. Descriptive statistics were used to describe the characteristics of the infants in both the exposed and non‐exposed cohorts (e.g., number of infants, birth weight, and gestational age). To evaluate an association between perinatal exposure to SARS-CoV-2 infection and early health outcomes, conditional logistic regression models (for binary outcomes) and linear mixed effects models (for continuous outcomes) were applied to the matched data. Additional analyses with effect modification by maternal residential area were performed, by including an interaction term of residential area with perinatal SARS-CoV-2 exposure to the models. As such, we examined whether associations between the perinatal SARS-CoV-2 exposure and the outcomes differed by residential area (rural vs. urban). Results were summarized using odds ratios (logistic models) and mean differences (linear models) and corresponding 95% confidence intervals. All analyses were conducted by using SAS 9.4 (SAS Institute Inc., Cary, NC). The current study was mainly for exploratory purposes, and no adjustment for multiple comparisons was performed.

## 3. Results

**Figure 1:**
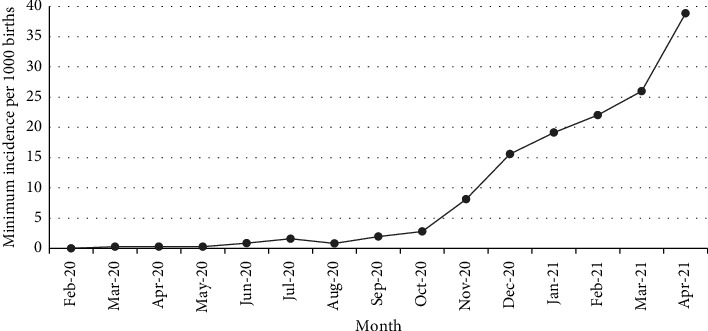
Monthly minimum incidence rates of infants born to COVID-19-positive mothers by total births in British Columbia, February 1, 2020, to April 30, 2021.

From February 1, 2020, to April 30, 2021, there were 52,711 live births in British Columbia. Among them, 484 were born to mothers positive for SARS-CoV-2 during pregnancy or at time of delivery, i.e., with perinatal exposure (9.18 per 1000 births). The number of infants born with perinatal exposure to SARS-CoV-2 markedly increased from November 2020 to April 2021 (Figure 1). Four infants with perinatal exposure to SARS-CoV-2 were excluded from the final analysis because of no matched control. As a result, 480 infants were included in the exposed cohort, whereas 1904 infants were included in the non‐exposed cohort.

Infants in the exposed cohort had baseline characteristics similar to infants in the non‐exposed cohort, with a mean gestational age of 38.5–38.7 weeks and mean birth weight of 3267–3336 grams (Table 1).

### 3.1. Perinatal Exposure to SARS-CoV-2 Infection, ED Visits, and Hospitalizations

We found that a significantly higher proportion of infants in the exposed cohort required at least one hospitalization (OR: 1.63; 95% CI: 1.11, 2.41) and at least one ED visit (OR: 1.40; 95% CI: 1.05, 1.85) when compared with those in the non‐exposed cohort during their first year of life (Table 2). The most common reasons for the infants in the exposed cohort to visit the ED were nausea/vomiting, feeding difficulties, fever, and rash (Table 2). The most common primary diagnoses for the infants in the exposed cohort to be hospitalized were neonatal jaundice, transient tachypnoea of the newborn, urinary tract infection, and COVID-19 (Table 2).

### 3.2. Perinatal Exposure to SARS-CoV-2 Infection and Infant Outcomes

The exposed cohort had a higher proportion of infants with a diagnosis in the category of “respiratory infectious diseases” compared with the non‐exposed cohort (OR: 1.62; 95% CI: 1.00, 2.63; Table 3. A significantly higher proportion of infants in the exposed cohort had a diagnosis in the category of “endocrine disorders” (OR: 2.67; 95% CI: 1.09, 6.52), which entailed mainly the diagnosis of hypoglycemia (Table 3).

### 3.3. Perinatal Exposure to SARS-CoV-2 Infection and Outpatient Medication Prescriptions

Infants in the exposed cohort were less likely to have outpatient medication prescriptions compared with unexposed infants (OR: 0.66; 95% CI: 0.44, 0.98).

### 3.4. Effect Modification by Mother's Residences

The effect of maternal residence (rural vs. urban) upon the association between SARS-CoV-2 exposure and early health outcomes/healthcare resource utilization was examined among the cases and matched controls (Supplementary Table 3). SARS-CoV-2-exposed infants in urban areas were significantly more likely to have a diagnosis of respiratory infectious diseases compared to the non‐exposed infants (OR: 1.74; 95% CI: 1.07, 2.84). Among urban infants, the associations between SARS-CoV-2 exposure and other outcomes remained consistent with the overall case-control analysis results (Supplementary Table 4). We could not compare urban vs. rural outcomes among the exposed infants, as the rural group was too small to estimate.

## 4. Interpretation

To our knowledge, this is one of the very few studies focusing on the healthcare resource utilization and early outcomes of infants born to mothers with a positive test of SARS-CoV-2 during pregnancy or at the time of delivery. We found a higher proportion of healthcare resource utilization, in terms of ED visits and hospitalizations, among infants with perinatal exposure to SARS-CoV-2 infection. The reasons for this association are likely multifactorial.

More frequent healthcare use in the exposed cohort can result from increased health concerns for infants with perinatal exposure to SARS-CoV-2. During the pandemic, little was known about the long-term health outcomes following potential exposure around the perinatal period. Thus, the perinatal exposure to SARS-CoV-2 infection in infants may have heightened the awareness of caregivers and parents, such that infants were more likely to be brought to the ED for medical assessment. History of perinatal SARS-CoV-2 exposure may have resulted in a lower threshold for hospital admission. On the other hand, the increased hospitalization may reflect potential changes in the fetus' developing immune system associated with SARS-CoV-2 infections during pregnancy. It is unclear whether in utero or perinatal exposure to maternal SARS-CoV-2 infections affects the developing immune system in neonates leading to adverse health outcomes in early infancy, and further research is needed to clarify this issue.

In this study, we found an elevated risk of hypoglycemia among infants in the exposed cohort. Although it is not clear whether the perinatal exposure to SARS-CoV-2 is a primary or secondary cause for the hypoglycemia, two possible hypotheses may explain this observation. First, the hypoglycemia could be secondary to feeding problems. We found that the most common clinical presentation of this group of exposed infants in ED visit was nausea/vomiting, feeding difficulties, fever, and rash. It is possible that the decreased oral intake, increased losses through vomiting, or increased metabolic demands from fever and/or infection could lead to hypoglycemia. Second, the hypoglycemia can also be related to an underlying disorder such as metabolic conditions, issues with liver synthetic function, or endocrine disorders. Reports in adults showed that SARS-CoV-2 infection can cause central hypocortisolism which resolves around three months after recovery from COVID-19 [21]. The mechanism was thought to be related to the cell destruction after the binding of SARS-CoV-2 viruses to the angiotensin-converting enzyme 2 (ACE2) receptors which are also found in the hypothalamus and pituitary cells [21, 22]. In infants whose mother who had COVID-19 during the pregnancy, the period of fetal pituitary gland development may be a period of particular susceptibility. Our 1-year follow-up duration did include the diagnoses coded as disorders of the adrenal or pituitary glands, but the number of total patients in both groups with these diagnoses was so few in the first 12 months of life that the association in this small cohort needs to be further evaluated. Hypoglycemia in exposed infants may also originate in maternal chronic or gestational diabetes as these mothers may be more likely to experience more severe symptoms of SARS-CoV-2 infection [23]. Interestingly, a case report suggested that SARS-CoV-2 infection can cause hyperglycemia in preterm infants [24]. Similarly, reports on adults also found increased risk of hyperglycemia and diabetes mellitus after SARS-CoV-2 infection, presumably due to the cell destruction in the pancreas after SARS-CoV-2 binding to ACE2 receptors in the pancreas [25]. The contradictory results indicate that further studies are needed to delineate the relationship between glycemic control and perinatal SARS-CoV-2 exposure.

We found that infants living in urban areas in the exposed cohort were more likely to have respiratory infections. Respiratory infections can spread more readily in the relatively crowded living environment which is common to urban areas. This finding could also be reflective of families living in rural communities, in general, who have limited access to healthcare services, and therefore the respiratory problem would be less likely to be diagnosed.

The strengths of this study include leveraging an interdisciplinary and well-established provincial administrative database to answer urgent questions during the early stages of the pandemic. We were able to integrate health sector and pharmacy data to understand how perinatal exposure has impacted longitudinal health outcomes. We were able to explore the longitudinal outcomes and geographic variations of early health indicators in the vast province of BC due to the superiority and availability of the database (i.e., linking COVID-19 testing results and perinatal information with prescription medications and health system interactions). Further, our study has the largest number of infants born to mothers with prior exposure to SARS-CoV-2 during the pregnancy, as compared to other similar studies in the literature currently.

### 4.1. Limitations

First, our study does not have information on the infant SARS-CoV-2 positivity status. This study was launched initially at the time when there were limited cases of COVID-19 in BC. As vertical transmission of SARS-CoV-2 to infants had been known to be rare [26], most hospital policies on SARS-CoV-2 testing of infants were limited to symptomatic neonates. We believe that this limitation does not have any significant impact on the validity of our results because the positivity rate of infants born to mothers with SARS-CoV-2 infection during pregnancy was low as shown in previous cohort studies [5–13]. Even the largest cohort (with 255 neonates born to 250 mothers) in Massachusetts showed that the neonatal positivity rate (as measured by a SARS-CoV-2 test at 24 hours and 48 hours of life) was only 2.2% and for those positive infants, half of them were asymptomatic [5]. Second, due to the data availability and updates, some infants born during the later months of 2021 had less than 12 months of follow-up. This affects both cohorts nondifferentially and thus may bias our results towards the null, and some differences between the cohorts may be underrepresented. Third, as an administrative data source, the information was not collected primarily for research purposes, and thus the research scope and depth can be affected. For example, our data did not include the stage of pregnancy or gestational age of maternal COVID-19 positive test, which may have affected the neonatal outcomes in various ways (i.e., infection in early pregnancy with transplacental protective antibodies vs. infection close to the time of delivery). We were not able to control the maternal health status before pregnancy in evaluating the neonatal outcomes. We were not able to discern any other infections or co-infections caused by other pathogens. Lastly, the non‐exposed group may have consisted of infants born to mothers with negative SARS-CoV-2 test results or to mothers who were not tested which may lead to differential misclassification of exposure status. However, during the period of the study, PCR testing of symptomatic individuals was provided routinely across the province which captured most COVID-19 cases. Therefore, most pregnant women would have either a positive or negative test result, and very few were not tested.

## 5. Conclusion

Our study provides insight into health outcomes and healthcare utilization of infants with perinatal exposure to SARS-CoV-2 by showing increased hospitalization and ED visits during their first year of life. A systematic and integrated approach to the surveillance of outcomes following perinatal exposure to SARS-CoV-2 infection is needed to describe the impact of COVID-19 on this population both during infancy and beyond. The impact of newer COVID variants on childhood outcomes after perinatal exposure need to be further studied.

## Figures and Tables

**Table 1:** Characteristics of infants born to mothers with a positive SARS-CoV-2 test (exposed cohort) and to mothers without (non‐exposed cohort).

	Infants in exposed cohort^*∗*^ (*n* = 480)	Infants in non-exposedcohort (*n* = 1904)	Mean difference or odds ratio (95% CI)^†^
Sex^*∗∗*^ (male); *n* (%)	262 (54.58%)	1037 (54.46%)	
Birthplace^*∗∗*^ (hospital); *n* (%)	475 (99.20%)	1884 (99.16%)	
Gestational age^*∗∗*^ (weeks); mean (SD)	38.51 (1.69)	38.66 (1.48)	
Preterm (<37 weeks GA); *n* (%)	44 (9.17%)	122 (6.41%)	1.79 (1.09, 2.92)
Birth weight (grams); mean (SD)	3267.21 (536.39)	3336.62 (501.66)	−66.24 (−111.63, −20.86)
Small-for-gestational age; *n* (%)	49 (10.21%)	163 (8.56%)	1.22 (0.87, 1.71)
Apgar score at 5 min; mean (SD)	8.90 (0.73)	8.90 (0.75)	−0.00 (−0.07, 0.07)
Maternal residence (rural); *n* (%)	26 (5.42%)	76 (3.99%)	1.49 (0.86, 2.56)

^*∗*^Only 480 out of 484 cases had matched controls. ^*∗∗*^Matching variable. ^†^The effect estimate for all outcomes was obtained by comparing exposed vs. non‐exposed cohorts. The effect estimate in the conditional logistic regression model (for binary outcomes) was presented as odds ratio (OR). The effect estimate in the linear mixed effect model (for continuous outcomes) was presented as regression coefficient (mean difference between the two groups).

**Table 2:** Healthcare resource utilization and reason for emergency department visit or hospitalization in the first 12 months after birth among infants with and without perinatal exposure to SARS-CoV-2.

	Infants in exposed cohort (*n* = 480)	Infants in non‐exposedcohort (*n* = 1904)	Odds ratio (95% CI)^†^
≥1 emergency department visit	81 (16.88%)	246 (12.92%)	**1.40 (1.05, 1.85)**
Nausea and/or vomiting	13 (2.71%)	32 (1.68%)	
Feeding difficulties in newborn	9 (1.88%)	16 (0.84%)	
Fever	9 (1.88%)	33 (1.73%)	
Rash	8 (1.67%)	10 (0.53%)	
Inconsolable crying	7 (1.46%)	24 (1.26%)	
Shortness of breath	7 (1.46%)	24 (1.26%)	
Neonatal jaundice	6 (1.25%)	29 (1.52%)	
≥1 hospitalization	39 (8.13%)	98 (5.15%)	**1.63 (1.11, 2.41)**
Neonatal jaundice	9 (1.89%)	29 (1.52%)	
Transient tachypnoea of the newborn	<5	5 (0.26%)	
Urinary tract infection	<5	<5	
COVID-19	<5	<5	

*n* values < 5 not reported. ^†^The effect estimate for all outcomes was obtained by comparing exposed vs. non‐exposed cohorts. Significant odds ratio (i.e., does not cross 1.0 in 95% CI).

**Table 3:** Early health outcomes and prescription medication needs in the first 12 months after birth among infants with and without perinatal exposure to SARS-CoV-2.

		Infants in exposed cohort (*n* = 480)	Infants in non‐exposedcohort (*n* = 1904)	Odds ratio (95% CI)^†^
*≥1 diagnosis (in- and outpatient combined)*				
Any		78 (16.25%)	293 (15.39%)	1.08 (0.81, 1.43)
General conditions (failure-to-thrive and infantile colic)		<5	<5	Unable to estimate
Infectious diseases		42 (8.75%)	138 (7.25%)	1.23 (0.86, 1.78)
Respiratory		25 (5.21%)	63 (3.31%)	1.62 (1.00, 2.63)
Bacterial (non‐respiratory)		19 (3.96%)	67 (3.52%)	1.14 (0.68, 1.92)
Viral (non‐respiratory)		<5	12 (0.63%)	1.00 (0.28, 3.62)
Allergic diseases		28 (5.83%)	125 (6.57%)	0.88 (0.57, 1.36)
Atopic dermatitis		21 (4.38%)	108 (5.67%)	
Allergic rhinitis		<5	11 (0.58%)	
Anaphylaxis		<5	10 (0.53%)	
Endocrine disorders		8 (1.67%)	12 (0.63%)	**2.67 (1.09, 6.52)**
Hypoglycemia		7 (1.46%)	8 (0.42%)	
Renal conditions		<5	17 (0.89%)	0.46 (0.11, 2.00)
Other diseases (conjunctivitis)		12 (2.50%)	32 (1.68%)	1.54 (0.77, 3.08)

*≥1 drug type by therapeutic class (outpatient only)*				
Any		32 (6.67%)	184 (9.66%)	**0.66 (0.44, 0.98)**
Eye, ear, nose, and throat preparations	Anti-infectives	9 (1.88%)	27 (1.42%)	1.35 (0.62, 2.93)
Skin and mucous membrane preparations	Anti-infectives	8 (1.67%)	51 (2.68%)	0.61 (0.28, 1.31)
	Anti-inflammatory agents	13 (2.71%)	88 (4.62%)	0.57 (0.31, 1.04)
Unclassified therapeutic agents		<5	22 (1.16%)	0.55 (0.16, 1.82)

*n* values < 5 not reported. ^†^The effect estimate for all outcomes was obtained by comparing exposed vs. non‐exposed cohorts. Significant odds ratio (i.e., does not cross 1.0 in 95% CI).

## Data Availability

Data are available on request. Data can be requested for research projects but are subject to approval through the Population Data BC Data Stewards.

## Supplementary Materials

Supplementary Materials Supplementary tables include the following: Supplementary Table 1. Selected diagnosis codes, descriptions and categories used to report early health outcomes. Supplementary Table 2. Selected Pharmanet drug types used by the patients in the analysis cohort. Supplementary Table 3. Distribution of infants in exposed and non‐exposed cohorts by rural vs. urban maternal residence. Supplementary Table 4. Effects of mother's residence (rural vs. urban) upon association between early health outcomes/ healthcare resource utilization and being born to mothers with a positive test of COVID-19 during pregnancy or at the time of delivery.
