# Supplementary material for: Impact of Perinatal Exposure to SARS-CoV-2 Infection on Early Health Outcomes among Infants Born from 2020 to 2021 in British Columbia, Canada
Source: Can J Infect Dis Med Microbiol. 2023 May 5;2023:9968774. doi: 10.1155/2023/9968774 (PMC10181900; doi:10.1155/2023/9968774)
Supplement: Supplementary Materials — Supplementary tables include the following: Supplementary Table 1. Selected diagnosis codes, descriptions and categories used to report early health outcomes. Supplementary Table 2. Selected Pharmanet drug types used by the patients in the analysis cohort. Supplementary Table 3. Distribution of infants in exposed and non‐exposed cohorts by rural vs. urban maternal residence. Supplementary Table 4. Effects of mother's residence (rural vs. urban) upon association between early health outcomes/ healthcare resource utilization and being born to mothers with a positive test of COVID-19 during pregnancy or at the time of delivery. [file 9968774.f1.docx]

**Supplementary Tables.**

**Supplementary Table 1.** Selected diagnosis codes, descriptions and categories used to report early health outcomes.

| **Category** | **Description** | **ICD10** | **ICD9** |
| --- | --- | --- | --- |
| Allergic Diseases | Allergic rhinitis | J30.1 | 477 |
|  | Allergy | T78.4; Z88; Z91.0 |  |
|  | Anaphylaxis | T78.0; T78.2 | 995 |
|  | Asthma | J45 | 493 |
|  | Atopic dermatitis | L20 | 691 |
|  | Conjunctivitis, allergic (other chronic) | H10.45 | 372.14 |
|  | Contact dermatitis | L23 | 692.9 |
|  | Eosinophilic esophagitis | K20.0 | 530.13 |
|  | Skin testing, scratch or intracutaneous tests |  | S00762; S00763; S00765 |
| Endocrine Disorders | Diabetes mellitus | E10; E11; E13; E14 | 250 |
|  | Disorders of adrenal glands |  | 255; 255.4; 255.5 |
|  | Disorders of lipid and mineral metabolism |  | 272; 275 |
|  | Disorders of parathyroid gland |  | 252 |
|  | Disorders of the pituitary gland |  | 253; 253.2; 253.5 |
|  | Hypoglycemia |  | 251.1; 251.2 |
|  | Hypothyroidism | E00; E01; E02; E03 | 243; 244 |
|  | Vitamin D deficiency |  | 268 |
| General Conditions | Failure-to-thrive | R62.51 | 783.41 |
|  | Infantile colic | R10.9 | 789.7 |
| Infectious Diseases  (Bacterial) | Abscess of skin |  | 680.9 |
|  | Bacterial infection of unspecified site | A49 | 041; 041.0; 041.1; 041.2; 041.3; 041.4; 041.5; 041.6; 041.7 |
|  | Boil |  | 041.0; 041.12; 041.11; 680.9 |
|  | Cellulitis | L03 | 682 |
|  | Empyema |  | 510 |
|  | Erysipelas |  | 035 |
|  | Escherichia coli | A04.0; A04.1; A04.2; A04.2; A04.3; A04.4; B96.2 | |
|  | Haemophilus influenzae | A49.2; B96.3 |  |
|  | Invasive group A streptococcus |  | 041 |
|  | Klebsiella pneumoniae | B96.1 |  |
|  | Mastoiditis |  | 383 |
|  | Meningococcal infection | A39 | 036 |
|  | MRSA colonization |  | V02.54 |
|  | Mycobacteria | A15; A31 | 031; 031.9 |
|  | Mycoplasma pneumoniae | A49.3; B96.0 |  |
|  | Proteus (mirabilis)(morganii) | B96.4 |  |
|  | Pseudomonas (aeruginosa) | B96.5 |  |
|  | Pyelonephritis | N10; N11; N12 | 040; 590.80 |
|  | Septicaemia |  | 038; 038.0; 038.1; 038.2; 038.3; 038.4; 038.8; 038.9 |
|  | Shigellosis |  | 004 |
|  | Streptococcus and staphylococcus | A49.0; A49.1; B95; B95.0; B95.1; B95.3; B95.6; U82.1 | |
|  | Typhoid and paratyphoid fevers | A01 | 002 |
|  | Urinary tract infection | N39.0 | 599 |
| Infectious Diseases  (Respiratory) | Acute bronchiolitis | J21 | 466.1 |
|  | Acute bronchitis | J20 | 466.0; 490 |
|  | Acute laryngitis and tracheitis | J04 | 464 |
|  | Acute nasopharyngitis (common cold) |  | 460 |
|  | Acute pharyngitis | J02 | 462 |
|  | Acute sinusitis | J01 | 461 |
|  | Acute tonsillitis | J03 | 463 |
|  | Croup |  | 464.4 |
|  | Influenza | J09; J10; J11 | 487 |
|  | Measles | B05 | 055; 484.0 |
|  | Pneumonia | J12; J13; J14; J15; J16; J17; J18 | 480; 480.0; 480.1; 480.2; 481; 482; 482.0; 482.2; 482.3; 482.4; 482.8; 485; 486 |
|  | Tuberculous |  | 010; 011 |
|  | Upper respiratory infection | J00; J01; J02; J03; J04; J05; J06 | 465 |
|  | Whooping cough | A37; A37.0 | 033; 484.3 |
| Infectious Diseases (Viral) | Adenovirus |  | 079 |
|  | Cholera |  | 001 |
|  | Coxsackie virus |  | 074; 079.2 |
|  | Cytomegalovirus |  | 078.5 |
|  | Echo virus |  | 079.1 |
|  | Enterovirus | B34.1; B97.1 |  |
|  | Erythema infectiosum | B08.3 |  |
|  | Gastroenteritis | A00; A02; A03; A04; A05; A06; A07; A08; A09 | 558.9 |
|  | Hand-Foot-and-Mouth disease | B08.4 | 078.4 |
|  | Hepatitis |  | 070; 070.1; 070.3; 070.7; 573.3 |
|  | Herpes simplex | B00 | 054; 054.4 |
|  | Herpes zoster |  | 053 |
|  | Herpetic Whitlow |  | 054.6 |
|  | Infectious mononucleosis |  | 075 |
|  | Respiratory syncytical virus | B97.4 |  |
|  | Rheumatic fever |  | 390 |
|  | Rhinovirus |  | 079.3 |
|  | Rubella |  | 056 |
|  | Varicella (chickenpox) | B01 | 052 |
| Infectious Diseases | Endocarditis | I33; I38; I39 |  |
|  | Meningitis | G00; G01: G02; G03 | 320.00; 047 |
|  | Otitis media | H65; H66; H67 |  |
|  | Sepsis | A40; A41 |  |
| Renal Conditions | Acidosis | N25.8 | 588.8 |
|  | Acute renal failure | N17.9 | 584.9 |
|  | Calculus | N20.0 | 592 |
|  | Chronic kidney disease | N18.9 | 585 |
|  | Electrolyte imbalance | E87.8 | 276.9 |
|  | Enuresis | R32 | 788.3 |
|  | Glomerulonephritis | N04.1; N05.9 | 581.3; 583.9 |
|  | Hematuria | N02.9 | 599.7 |
|  | Hydronephrosis | N13.3 | 591 |
|  | Hypercalciuria | E83.5 | 275.49 |
|  | Hypertension | I12 | 403.9 |
|  | Nephrocalcinoisis | E83.51 | 275.49 |
|  | Nephrotic syndrome | N04.9 | 581.9 |
|  | Proteinuria | N39.1 | 791 |
|  | Urinary tract malformations | Q64.9; Q62.0; Q62.10; Q64.2; Q61.4; Q61.9; Q60.0 | 753.9; 753.29; 753.21; 753.8; 753.15; 753.13; 753.16; 753.0 |
| Respiratory Conditions | Dependence on supplemental oxygen | Z99.81 | V46. 2 |
| Other | Conjunctivitis | B30; H10 | 372 |

**Supplementary Table 2.** Selected Pharmanet drug types used by the patients in the analysis cohort.

| **TC1** | **TC2** |
| --- | --- |
| ANTI-INFECTIVE | AMBECIDES |
| ANTI-INFECTIVE | ANTIBIOTICS |
| ANTI-INFECTIVE | ANTIMALARIAL AGENTS |
| ANTI-INFECTIVE | ANTITUBERCULOSIS AGENTS |
| ANTI-INFECTIVE | ANTIVIRALS |
| ANTI-INFECTIVE | MISCELLANEOUS ANTI-INFECTIVES |
| ANTI-INFECTIVE | QUINOLONES |
| ANTI-INFECTIVE | SULFONAMIDES |
| ANTI-INFECTIVE | URINARY ANTI-INFECTIVES |
| ANTIHISTAMINES | ANTIHISTAMINE & OTHER DRUGS |
| ANTIHISTAMINES | ANTIHISTAMINES |
| ANTIHISTAMINES | UNKNOWN |
| ANTINEOPLASTIC | ANTINEOPLASTIC |
| ANTITUSSIVES,EXPECTOR,MUCOLYT AGEN | ANTITUSSIVES |
| ANTITUSSIVES,EXPECTOR,MUCOLYT AGEN | EXPECTORANTS |
| ANTITUSSIVES,EXPECTOR,MUCOLYT AGEN | UNKNOWN |
| AUTONOMIC | ADRENERGIC AGENTS |
| AUTONOMIC | ANTICHOLINERGIC AGENTS |
| CARDIOVASCULAR | ANTILIPEMIC AGENTS |
| CARDIOVASCULAR | CARDIAC DRUGS |
| CARDIOVASCULAR | HYPOTENSIVE AGENTS |
| CENTRAL NERVOUS SYSTEM | ANALGESICS & ANTIPYRETICS |
| CENTRAL NERVOUS SYSTEM | ANTICONVULSANTS |
| CENTRAL NERVOUS SYSTEM | ANXIOLYTICS SEDS & HYPNOTICS |
| CENTRAL NERVOUS SYSTEM | PSYCHOTHERAPEUTIC AGENTS |
| DIAGNOSTIC AGENTS | THYROID FUNCTION |
| DIAGNOSTIC AGENTS | TUBERCULOSIS |
| ELECTROLYTIC,CALORIC,WATER BALANCE | ALKALINIZING AGENTS |
| ELECTROLYTIC,CALORIC,WATER BALANCE | CALORIC AGENTS |
| ELECTROLYTIC,CALORIC,WATER BALANCE | DIURETICS |
| ELECTROLYTIC,CALORIC,WATER BALANCE | IRRIGATING SOLUTIONS |
| ELECTROLYTIC,CALORIC,WATER BALANCE | MISC. ELECTROLYTIC, CALORIC & W.B. |
| ELECTROLYTIC,CALORIC,WATER BALANCE | POTASSIUM REMOVING RESINS |
| ELECTROLYTIC,CALORIC,WATER BALANCE | REPLACEMENT SOLUTIONS |
| EYE, EAR,NOSE, THROAT PREPARATION | ANTI-INFECTIVES |
| EYE, EAR,NOSE, THROAT PREPARATION | ANTI-INFLAMMATORY AGENTS |
| EYE, EAR,NOSE, THROAT PREPARATION | MISC E.E.N.T. DRUGS |
| EYE, EAR,NOSE, THROAT PREPARATION | VASOCONSTRICTORS |
| GASTROINTESTINAL | ANTACIDS & ADSORBENTS |
| GASTROINTESTINAL | ANTIDIARRHEA AGENTS |
| GASTROINTESTINAL | ANTIEMETICS |
| GASTROINTESTINAL | CATHARTICS & LAXATIVES |
| HORMONES & SYNTHETIC SUBSTITUTES | ADRENALS |
| HORMONES & SYNTHETIC SUBSTITUTES | ANDROGENS |
| HORMONES & SYNTHETIC SUBSTITUTES | ANTIDIABETIC AGENTS |
| HORMONES & SYNTHETIC SUBSTITUTES | ESTROGENS |
| HORMONES & SYNTHETIC SUBSTITUTES | GONADOTROPINS |
| HORMONES & SYNTHETIC SUBSTITUTES | PARATHYROID |
| HORMONES & SYNTHETIC SUBSTITUTES | PITUITARY |
| HORMONES & SYNTHETIC SUBSTITUTES | THYROID & ANTITHYROID AGENTS |
| HORMONES & SYNTHETIC SUBSTITUTES | UNKNOWN |
| NON-DRUG ITEMS | NON-DRUG ITEMS |
| SKIN & MUCOUS MEMBRANE PREPS | ANTI-INFECTIVES |
| SKIN & MUCOUS MEMBRANE PREPS | ANTI-INFLAMMATORY AGENTS |
| SKIN & MUCOUS MEMBRANE PREPS | ANTIPRURITICS & LOCAL ANESTHETICS |
| SKIN & MUCOUS MEMBRANE PREPS | CELL STIMULANTS AND PROLIFERATIONS |
| SKIN & MUCOUS MEMBRANE PREPS | EMOLLIENTS,DEMULCENTS & PROTECTANT |
| SKIN & MUCOUS MEMBRANE PREPS | KERATOLYTIC AGENTS |
| SKIN & MUCOUS MEMBRANE PREPS | MISC SKIN & MUCOUS MEMBRANE AGENTS |
| SMOOTH MUSCLE RELAXANTS | RESPIRATORY S M R |
| UNCLASSIFIED THERAPEUTIC AGENTS | ALLERGENIC EXTRACTS |
| UNCLASSIFIED THERAPEUTIC AGENTS | CMPS/CORR/INVEST |
| UNCLASSIFIED THERAPEUTIC AGENTS | UNCLASSIFIED THERAPEUTIC AGENTS |
| VITAMINS | VITAMIN D |

**Supplementary Table 3.** Distribution of infants in exposed and non-exposed cohorts by rural vs. urban maternal residence.

|  | **Maternal Residence** | | **TOTAL** |
| --- | --- | --- | --- |
|  | **Urban** | **Rural** |  |
| **Infants in exposed cohort** | 454 (94.5%) | 26 (5.4%) | 480 |
| **Infants in non-exposed cohort** | 1828 (96.0%) | 76 (4.0%) | 1904 |
| **TOTAL** | 2282 (95.7%) | 102 (4.3%) | 2384 |

**Supplementary Table 4.** Effect of mother's residence (rural vs. urban) upon association between early health outcomes/ healthcare resource utilization and being born to mothers with a positive test of COVID-19 during pregnancy or at the time of delivery.

|  | | **Odds Ratio (95% CI)†**  **Stratified by Maternal Residence** | | **Likelihood Ratio Test***  p-value |
| --- | --- | --- | --- | --- |
|  |  | **Urban (n=2282)** | **Rural (n=102)** |  |
| **Healthcare resource** | | | | |
| Emergency department visit | | **1.41 (1.06, 1.88)** | Unable to estimate | n/a |
| Hospitalization | | **1.59 (1.07, 2.37** | 2.94 (0.31, 28.09) | 0.60 |
| **Diagnosis** | | | | |
| Any | | 1.13 (0.85, 1.51) | Unable to estimate | n/a |
| General conditions (failure-to-thrive and infantile colic) | | Unable to estimate | Unable to estimate | n/a |
| Infectious diseases | | 1.29 (0.89, 1.87) | Unable to estimate | n/a |
| *Respiratory* | | **1.74 (1.07, 2.84)** | Unable to estimate | n/a |
| *Bacterial (non-respiratory)* | | 1.17 (0.70, 1.97) | Unable to estimate | n/a |
| *Viral (non-respiratory)* | | 1.05 (0.29, 3.88) | 1.05 (0.29, 3.88) | 1.00 |
| Allergic diseases | | 0.92 (0.59, 1.43) | Unable to estimate | n/a |
| Endocrine disorders | | **3.24 (1.27, 8.26)** | Unable to estimate | n/a |
| Renal conditions | | 0.46 (0.11, 2.01) | Unable to estimate | n/a |
| Other diseases (conjunctivitis) | | 1.58 (0.79, 3.17) | Unable to estimate | n/a |
| **Drug type by therapeutic class** | | | | |
| Any | | **0.66 (0.44, 0.99)** | Unable to estimate | n/a |
| Eye, ear, nose throat preparations | *Anti-infectives* | 1.34 (0.61, 2.95) | 1.34 (0.61, 2.95) | 1.00 |
| Skin and mucous membrane preparations | *Anti-infectives* | 0.60 (0.28, 1.30) | Unable to estimate | n/a |
|  | *Anti-inflammatory agents* | 0.59 (0.32, 1.07) | Unable to estimate | n/a |
| Unclassified therapeutic agents | | 0.52 (0.16, 1.74) | 0.52 (0.16, 1.74) | 1.00 |

*†The effect estimate for all outcomes were comparing exposed vs. non-exposed cohorts.*

**The likelihood ratio test (LRT) - a statistical test of the goodness-of-fit between two models, was run to compare the models with interaction terms (between exposure and maternal residence) vs. the models without.*
